# The Effect of Laponite on the Structure, Mechanical and Thermal Properties of Poly(butylene Succinate)

**DOI:** 10.3390/polym16223186

**Published:** 2024-11-16

**Authors:** Rafał Malinowski, Volodymyr Krasinskyi, Krzysztof Moraczewski, Aneta Raszkowska-Kaczor, Oleksandr Grytsenko, Volodymyr Moravskyi, Andrzej Miklaszewski

**Affiliations:** 1 Łukasiewicz Research Network—Institute for Engineering of Polymer Materials and Dyes, 55 M. Skłodowska-Curie St., 87-100 Toruń, Poland; volodymyr.krasinskyi@impib.lukasiewicz.gov.pl (V.K.); aneta.kaczor@impib.lukasiewicz.gov.pl (A.R.-K.); 2 Faculty of Materials Engineering, Kazimierz Wielki University, 30 Chodkiewicza St., 85-064 Bydgoszcz, Poland; kmm@ukw.edu.pl; 3 Department of Chemical Technology of Plastics Processing, Lviv Polytechnic National University, 12 Bandera St., 79013 Lviv, Ukraine; ogryts@gmail.com (O.G.); vmoravsky@gmail.com (V.M.); 4 Institute of Material Science and Engineering, Poznan University of Technology, 24 Jana Pawła II St., 60-965 Poznan, Poland; andrzej.miklaszewski@put.poznan.pl

**Keywords:** poly(butylene succinate), Laponite, composites, mechanical properties, thermal properties, extrusion

## Abstract

The comparison of some changes occurring in the physical properties of poly(butylene succinate) (PBS) due to its modification with Laponite (LAP) was the objective of this study. The PBS composites, containing from 1 to 7 wt% LAP, were prepared by co-rotating twin screw extrusion. The geometrical surface structure of the samples’ fractures and the LAP content in the individual composites were examined. In addition, changes in the mechanical and thermal properties, melt flow rate and moisture sorption of the composites were studied. It was found that LAP increases the elastic modulus of PBS and also decreases its impact strength and, in particular, its strain. The strength of PBS changes to a lesser extent with an increase in the LAP content, i.e., it slightly decreases in tensile tests or slightly increases in bending tests. LAP also lowers the flow rate of PBS and significantly increases moisture sorption. Moreover, the composites produced were characterized by a uniform distribution of the dispersed phase in the polymer matrix and an acceptable adhesion at the interface between the two components.

## 1. Introduction

Biodegradable polymer composites and their processing constitute the subject of numerous research studies related to the development and implementation of new environmentally friendly products. This may result from the fact that the biodegradable polymers themselves are often unable to be compared with their non-biodegradable equivalents. This refers to the properties of these polymers, as well as to their processing and pricing. Therefore, producing composites is one of the more common ways to modify the various properties of these biodegradable polymers. The composites whose dispersed phase consists of natural fillers (plant or mineral) may play an especially important role in many branches of economy [1,2,3]. One of the most commonly used fillers are natural clay minerals from the smectite group, the best-known representative of which is montmorillonite (MMT) [4,5,6,7,8].

**Figure 1:**
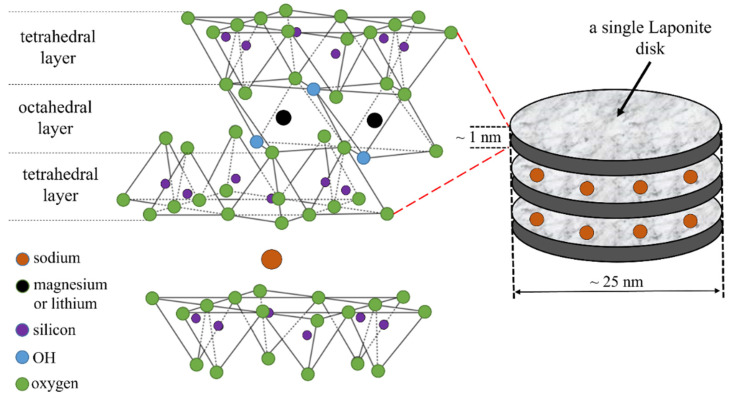
Structure of Laponite (LAP).

This group also includes a lesser-known natural mineral—hectorite (Htr) [9,10,11]. It is the subject of numerous research studies [12,13,14]. In this study, however, we will focus not on Htr but on its synthetic variety, Laponite (LAP) [15,16,17,18]. The name Laponite was introduced by the company Laponite Industries in the 1960s. This name is commonly used as a synonym for synthetic hectorite. LAP shows many valuable advantages compared to Htr, e.g., (a) it has high purity, (b) the properties of LAP can be tailored during synthesis for specific application needs, (c) it is possible to synthesize variants of LAPs that do not have natural equivalents and (d) larger production of LAP means at the same time less exploitation of limited natural deposits [19]. In a similar way to MMT, it is composed of two layers (octahedral and tetrahedral), type 2:1 [20]. However, unlike MMT, LAP is a smectite mineral with a trioctahedral structure [21]. This is mainly due to the fact that its octahedral layer is dominated by divalent magnesium cations. LAP forms nanometer-sized particles in the shape of a “disk” as shown in Figure 1. Moreover, as a consequence of isomorphic substitution (replacing some magnesium atoms with lithium atoms), small negative charges accumulate on the surface of the disks, and small positive charges accumulate on the edges. This causes the LAP disks to resemble small dipoles, which can form a spatial network by creating physical entanglements or cross-links between different polymer chains [22]. This process easily occurs in solutions where LAP forms a suspension. In turn, in polymer melts it is not easy because LAP tactoids are very difficult to disperse into individual disks during their processing with a specific polymer. Nevertheless, the use of LAP with strong hydrophilic properties may prove beneficial when modifying the properties of biodegradable polymers, which may thus acquire new and valuable functional properties.

Poly(butylene succinate) (PBS) is one of the well-known biodegradable polymers [23,24,25]. It belongs to the family of aliphatic biodegradable polyesters [26,27]. This polymer exhibits good biodegradability, including in soil, thermoplastic behavior, thermo-processability using conventional equipment for plastics, good chemical resistance and reasonably-balanced thermomechanical properties [28,29]. Moreover, PBS has received great interest due to its properties that are comparable to those of polyolefins [30,31]. Therefore, it may be a suitable replacement for non-biodegradable polyethylene (PE). PBS is a semi-crystalline thermoplastic polymer with a melting point (T_m_) in the range of 90–130 °C, and a low glass transition temperature (T_g_) of about −45 to −10 °C depending on its type [32]. PBS also has a wide temperature range; namely, it can even be processed at temperatures of up to 200 °C [33]. However, PBS is also characterized by some drawbacks, such as excessive softness, poor gas barrier properties, high crystallinity and high unit price, which may be a problem in some applications.

The studies published so far have demonstrated many benefits of using LAP to modify various properties of polymers. Zhou et al. [34] investigated laponite-poly(L-lactide) composite films prepared by the solvent method. The authors found that using LAP as a complexing agent can improve the mechanical properties and thermal stability of PLA. Wang et al. [35] constructed a biomimetic urethral repair substitute based on nano-Laponite/polylactic acid-glycolic acid copolymer fiber scaffolds. Liu et al. [36] carried out a study on the effects of LAP (0, 1, 2 and 3 wt%) on poly(lactic-acid)/poly(trimethylene-carbonate) composite film properties. They also investigated the effects of the use of such composite films on the postharvest quality of mushrooms (*Agaricus bisporus*). The results obtained confirmed the applicability of the LAP filler in the films studied and the use of these composite films in the packaging of the mushrooms. In turn, the effect of the LAP content on the mechanical and barrier properties of the starch/PVOH-based films was evaluated by Sharma et al. [37]. They found that up to 10 wt% of LAP filler improved the mechanical and barrier properties of the starch/PVOH films. Studies on polycaprolactone-laponite composite scaffold were also conducted [38]. The authors confirmed that an optimal composition of the scaffold with 3 wt% laponite-drug complex loading is ideal for obtaining enhanced alkaline phosphatase activity by maintaining cell viability. LAP was also used to produce cellulose-based composites for printing biodegradable printed circuit boards [39]. The addition of the LAP into the cellulose increased the degradation temperature and flame retardancy and decreased the mechanical properties of the cellulose-laponite composites. Other composites of LAP and natural polymers were studied by Gonzaga et al. [40]. The chitosan-laponite-based scaffolds they developed exhibited a porous architecture. Moreover, an increase in clay content led to an increase in porosity, an improvement in mechanical strength and a decrease in the swelling capacity.

Modification of PBS properties (a biodegradable polymer with properties equivalent to PE) with the use of LAP is so far unknown. Furthermore, the search for new biodegradable materials that could replace classical PE seems necessary. Therefore, the effects of such a modification may be interesting from cognitive and utilitarian viewpoints. This also refers to fully biodegradable films that could exhibit increased or decreased barrier properties upon the addition of LAP. In these films, LAP, in a modified or unmodified form, could be an alternative to the commonly used MMT. These were the main reasons why LAP was chosen for the study. The main objective of this study was to investigate the effect of the LAP on the changes in some properties of PBS. The changes in mechanical, thermal and rheological properties, as well as in the moisture sorption and in the structure of the produced composites, upon addition of various amounts of LAP were determined.

## 2. Materials, Methods and Characterization

### 2.1. Materials

The composite matrix used was poly(butylene succinate) (PBS), type BioPBS^TM^ FZ71PM (PTT MCC Biochem CO., Ltd., Bangkok, Thailand). Its melt flow rate was 22 g/10 min (2.16 kg, 190 °C) with a density of 1.26 g/cm^3^ and the melting point 115 °C. To modify its properties, non-intercalated Laponite (LAP), type Laponite-RD (Rockwood Additives Ltd., Widnes, UK) with the formula ${\mathrm{Na}}_{0.7}^{+}\left[({\mathrm{Si}}_{8}{\mathrm{Mg}}_{5.45}{\mathrm{Li}}_{0.3}\right)O_{20}{\left(\mathrm{OH}{)}_{4}\right]}^{-0.7}$, a density of 2.53 g/cm^3^ and a decomposition temperature above 500 °C was used. The modifier LAP was in the form of a white powder with particle sizes ranging from 5 to 100 μm. The particles were characterized by an irregular geometry and formed multi-walled solids (Figure 3).

### 2.2. Methods of Sample Preparation

**Figure 2:**
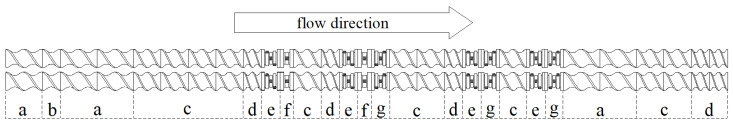
Design of screws of co-rotating twin screw extruder; a—conveying segments of 40-mm length and 40-mm pitch, b—conveying segments of 20-mm length and 40-mm pitch, c—conveying segments of 30-mm length and 30-mm pitch, d—conveying segments of 20-mm length and 20-mm pitch, e—kneading segments of 20-mm length, providing intensive distributive mixing, f—kneading segments of 15-mm length, providing intensive dispersive mixing and g—reverse kneading segments of 20-mm length, providing intensive distributive mixing.

The extrusion of the pristine PBS and PBS composites containing 1, 3, 5 and 7 wt% of LAP was carried out using a co-rotating twin screw extruder type BTSK 20/40D (Bühler, Braunschweig, Germany). The extruder was equipped with screws of 20-mm diameter with an L/D ratio of 40 and a two-opening die head. PBS was dosed directly to the extruder hopper feeder, while LAP was dosed directly to the barrel zone at L/D 12. The extrusion was performed maintaining the following temperatures of extruder zones: 150, 153, 156 and 159 °C. The temperature of the extrusion die-head was 160 °C. The screw rotation speed was constant, being equal to 150 rpm. The plasticizing system was equipped with one non-vacuum degassing zone at L/D 32. Prior to the extrusion, the PBS and LAP were dried for 24 h at 70 and 120 °C, respectively. The extruded composites were cooled intensively on a conveyor belt under an air stream and then granulated. The extrusion process was carried out with the use of a special shape of screws containing different segments. The screws were of a complex design; they consisted of conveying segments, reverse segments and kneading segments, including elements providing intensive dispersive mixing and intensive distributive mixing (Figure 2).

During the course of the extrusion, the basic parameters of this process were recorded. They included (i) the extruder motor torque (*M*), (ii) the energy consumption (*P*) by the extruder drive system and (iii) the temperature (*T_D_*) of the material being processed, measured in the extruder die-head. The granulates obtained were then processed to obtain standard specimens designed for mechanical and structural investigations. Standard dumbbell- and bar-shaped specimens were prepared according to a relevant standard (PN-EN ISO 527-2:1998) by using an injection molding machine (Battenfeld Plus 35/75, Solingen, Germany). The temperatures of the barrel plasticizing zones I and II were 155 and 160 °C, respectively, and that of the injection head, 165 °C. The injection mold temperature and the injection pressure were constant at 40 °C and 140 MPa, respectively.

### 2.3. Characterizations

The LAP structure and the geometrical surface structure of the sample fractures were determined by a scanning electron microscope (SEM), type Hitachi SU8010 (Hitachi, Tokyo, Japan). Previously injected bar-shaped specimens were used to make the fractures in liquid nitrogen. Imaging studies were performed at an accelerating voltage of 15 kV with the use of SE or BSE detectors. An X-ray microanalysis was carried out using an EDX detector and an accelerating voltage of 30 kV. The percentage composition of the LAP and the changes in the LAP content in the composites obtained were determined. Differential scanning calorimetry (DSC) was performed using a DSC 1 STARe System differential scanning calorimeter (Mettler Toledo, Greifensee, Switzerland). The DSC measurements were performed under nitrogen with a flow rate of 50 mL/min. The samples were successively heated from 10 to 160 °C at 10 °C/min, annealed at 160 °C for 3 min, cooled to 10 °C at 10 °C/min, and reheated to 160 °C at a rate of 10 °C/min. The cooling cycle and the second heating cycle were used to analyze the thermal properties of the samples studied. The tensile strength (σ_M_), longitudinal modulus of elasticity (E_t_) and elongation at break (ε_B_) were evaluated according to the PN-EN ISO 527-1:2020 standard, using the extension rate of 10.0 mm/min. The flexural stress at conventional deflection (σ_fc_) and flexural modulus (E_f_) were measured by the three-point bend test at the bending deflection rate of 5.0 mm/min. The measurements were carried out in accordance with the PN-EN ISO 178:2011/A1 standard. Mechanical properties tests under static tension and static three-point bending were carried out using a tensile testing machine, type TIRAtest 27,025 (TIRA Maschinenbau GmbH, Schalkau, Germany). The impact strength (a_cN_) was evaluated according to the PN-EN ISO 179-1:2010 standard and with the use of a pendulum Impact Tester, type IMPats-15 (ATS FAAR, Novegro-Tregarezzo, Italy). The melt flow rate (MFR) was measured according to the PN-EN ISO 1133:2020 standard (190 °C, 2.16 kg) by a capillary plastometer, type LMI 4003 (Dynisco, Franklin, TN, USA). The moisture content was determined using a MAX 50/1 moisture analyzer (RADWAG, Radom, Poland) in accordance with the PN-C 89,418 standard. In the static tensile, static three-point bending and MFR tests, five measurements were performed for each sample and then the arithmetic mean was calculated. In the impact tests, the arithmetic mean was calculated from ten measurements performed for each sample.

## 3. Results and Discussion

### 3.1. Microscopic Observation

**Figure 3:**
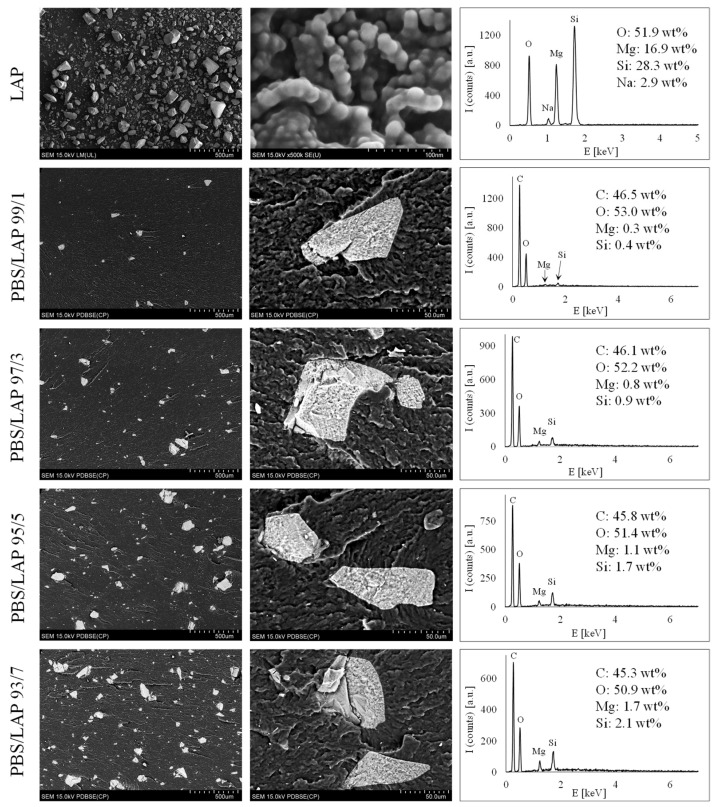
SEM images ((**left**) and (**middle**) column) and EDX microanalysis ((**right**) column) of LAP and PBS/LAP composites.

The microscopic observations of the filler’s structure (LAP) and the composites produced (PBS/LAP) are presented in Figure 3. The figure also shows the results of the X-ray microanalysis of these materials.

As seen in Figure 3, the particles of the filler used have very different sizes. The vast majority of them are between 10 and 100 µm in size [41]. Moreover, at a higher magnification, it can be seen that these particles are composed of nanometer-sized LAP tactoids. The LAP used is also characterized by a high purity, as shown in the EDX analysis. The SEM analysis of the PBS/LAP composites indicates three important issues. Firstly, quite large LAP particles are observed in the PBS matrix, whose sizes are similar to those of a pure filler. This means that this filler was not fragmented (dispersed) during the extrusion and represents a dispersed phase with micrometer-sized particles. Secondly, it can be observed that the distribution of the filler is quite good. The LAP particles are evenly distributed in the PBS matrix and do not form agglomerates. In the SEM images taken at a higher magnification (middle column), quite good adhesion at the interface between the PBS and LAP can also be observed. Usually, there are no free spaces there, which indicates a good compatibility of both phases. This may be partly due to the polar nature of both the PBS and the LAP, caused by the presence of numerous hydroxyl groups in the PBS and the electrical charges in the LAP [42,43]. Additional EDX microanalyses of individual composites show that the amounts of elements such as Mg and Si increase linearly with the rise in the LAP content in the PBS matrix. This proves that the composites studied were well manufactured and contain the amounts of filler that were originally expected. The most important information resulting from the SEM analyses refers to the size of the LAP particles that occur in the PBS matrix. It can be said that there are no nanometer-sized individual LAP disks or even their tactoids in this matrix. The presence of micrometric-sized, practically unchanged LAP particles may have a completely different effect on the thermal and mechanical properties of the composites studied than when particles of nanometric size occur in this type of composites.

### 3.2. Thermal Analysis

**Figure 4:**
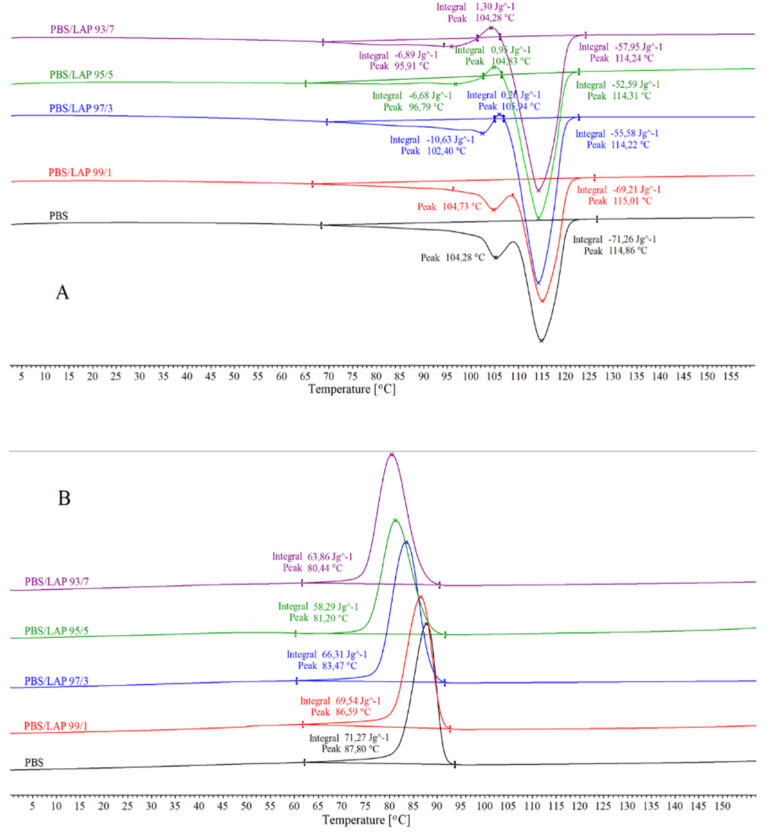
DSC curves recorded for samples studied ((**A**)—second heating; (**B**)—cooling).

The DSC results are presented in Figure 4 and summarized in Table 1. The results shown in Figure 4A clearly indicate that all the samples are characterized by the occurrence of two endothermic melting peaks, and some additionally by the presence of a single recrystallization peak before the final melting stage. There are several theories to explain the multiple melting behavior of semi-crystalline polymers, the most important of which is the presence of melting, recrystallization and re-melting phenomena [44,45,46]. According to this, the first stage is usually the melting of the original crystallites (lower melting crystallites with lower thermal stability) and their recrystallization into a more thermally stable form. The second stage involves melting the more perfect crystallites with higher thermal stability [47].

Such a situation is mainly observed in the PBS sample and the composite with the lowest LAP content; the thermal effects of these samples are similar. Both samples show an endothermic melting peak of the crystalline phase at ca. 105 °C. In these samples, after melting the crystallites at 105 °C, they recrystallize into a more stable crystallographic structure that melts again at 115 °C. It is worth noting here that the melting temperature of the more thermally stable crystallites is similar in all composites. Thus, it does not depend on the LAP content. However, the filler has a very significant effect on the formation of less thermally stable crystallites, i.e., those formed at temperatures from ca. 95 to ca. 105 °C. This can already be seen in the sample containing 3 wt% LAP. A small endothermic melting peak of the crystalline phase (ca. 103 °C) with an enthalpy of ca. 10.6 J/g, and a very small exothermic peak of cold crystallization (ca. 106 °C) with an enthalpy of ca. 0.3 J/g can be observed for this sample. In turn, in samples with the highest LAP content (5 or 7%), the first melting peaks of the crystalline phase almost completely disappear, and higher and higher peaks of cold crystallization appear (ca. 105 and ca. 104 °C for the samples containing 5 and 7 wt% LAP, respectively), which immediately melt at ca. 114–115 °C after crystallization. Thus, as the LAP content increases, the number of original crystallites, i.e., lower melting crystallites, decreases.

The thermal changes presented in the range of 95–106 °C are closely associated with the thermal effects observed during the melt-crystallization of the individual samples (Figure 4B). In this process, different types of crystals with different stabilities can be formed at the same time [48]. The decreasing melt-crystallization temperature with an increasing LAP content, and thus the decreasing growth rate of the crystalline phase, indicates that different types of crystallites may be formed in the composites studied in different amounts. This statement refers mainly to the crystallite fraction that is less thermally stable and forms in small amounts in composites with the highest LAP content. These types of crystallites, which were not formed, or were formed in slight amounts during the melt-crystallization, are fully formed during the cold crystallization in the second heating cycle, as can be seen in Figure 4A. In the composites with a low LAP content (1 or 3 wt%), both types of crystallites (i.e., with different crystallographic structures and thermal stabilities) are formed during the melt-crystallization process; however, the more thermally stable ones should be much more dominant which is also confirmed in the results in Figure 4A. Thus, taking into account the cooling and heating processes simultaneously, it can be concluded that the LAP with the structure shown in Figure 3 hinders the migration and reorientation of the PBS macromolecules, preventing or limiting the formation of crystallites with a lower thermal stability [49]. It can also be said that LAP does not cause the nucleation effect to occur. On the contrary, crystallites form in smaller quantities near the LAP particles. This may also be due to the large dimensions of the LAP particles used. The above statements are also confirmed by the values of the crystallization enthalpy and the melting enthalpy of the crystalline phases. The sum of the values of the melt-crystallization enthalpy and the enthalpy of the cold crystallization in each sample is equal to the sum of the melting enthalpy values of both types of crystallites (with lower and higher thermal stabilities).

### 3.3. Mechanical Properties

**Figure 5:**
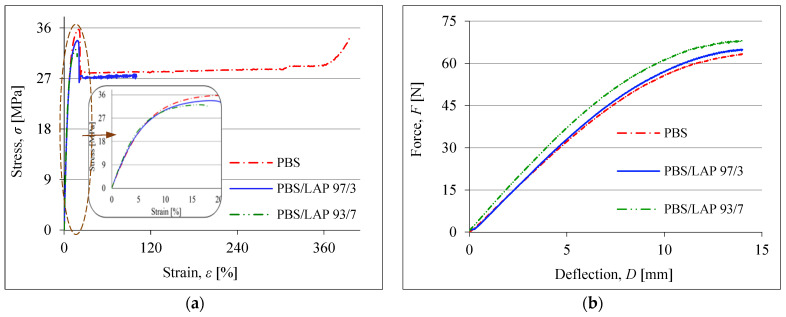
Static tension (**a**) and static three-point bending (**b**) curves of PBS and selected composites.

**Figure 6:**
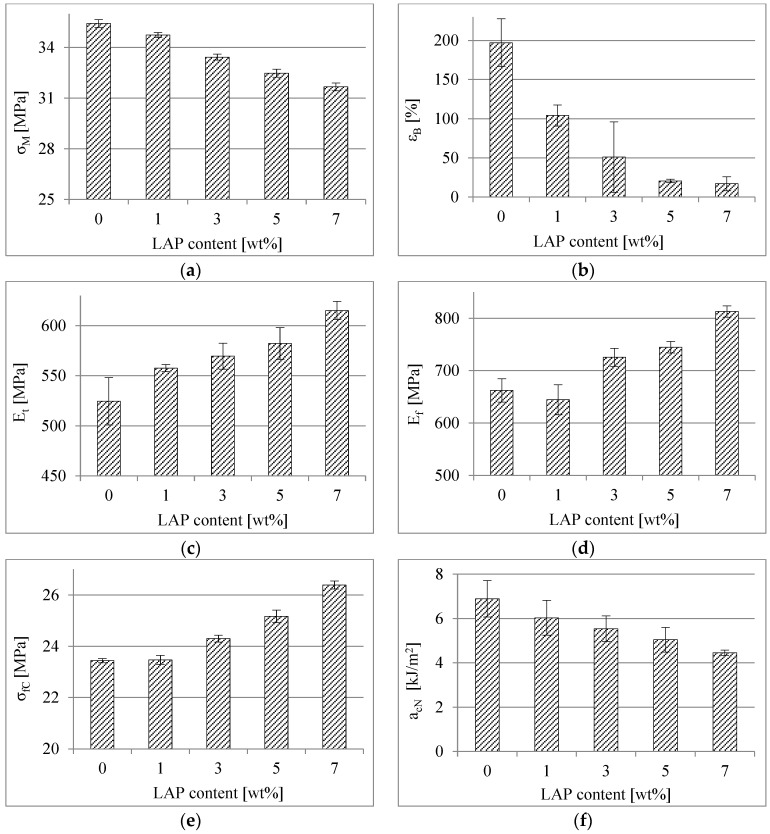
Mechanical properties results [(**a**)—tensile strength, (**b**)—elongation at break, (**c**)—longitudinal modulus of elasticity, (**d**)—flexural modulus, (**e**)—flexural stress at conventional deflection, (**f**)—impact strength].

The changes in the mechanical properties of the PBS after its modification with the LAP are presented in Figure 5 and Figure 6. Exemplary curves concerning the static tension tests and the static three-point bending tests are shown in Figure 5. Detailed changes in σ_M_, ε_B_, E_t_, E_f_, σ_fC_ and a_cN_ are presented in Figure 6.

Analyzing the data presented, it can be concluded that the LAP causes a decrease in the values of σ_M_, ε_B_ and a_cN_ of PBS, but at the same time increases E_t_, E_f_ and σ_fC_. In general, the changes in PBS’ mechanical properties caused by the introduction of the LAP into its matrix are quite significant. The composites obtained are mainly more rigid and have less deformation. Similar conclusions were also found by other researchers studying various LAP composites [50,51]. The greatest changes are observed in the elongation at break, where it decreased by almost 12 times after adding 7 wt% LAP. It is worth noting here that the ε_B_ value of the PBS containing 3 wt% LAP is characterized by a very large confidence interval. This indicates that some samples showed very low elongation and some showed very high elongation. This, in turn, may indicate the limit of significant deformation of the PBS determined by the presence of LAP in the matrix of this polymer. The decrease in impact strength (by about 35%) is also significant, but when a mineral filler with a micron grain size is used, this is a fairly common trend. Nevertheless, the composites obtained are characterized by good mechanical properties. Even if some of them have deteriorated, the materials made from this type of composite will still be able to be widely used, especially in packaging, including thermoforming of rigid packaging.

It is also worth noting that the basic processing parameters did not change significantly during the manufacturing of the materials studied (Table 2). The largest amount of LAP (7 wt%) increased the values of M, P and T_D_ by 15, 12 and 1%, respectively. Thus, the production of various products from this type of materials should not cause technological problems.

### 3.4. Mass Melt Flow Rate and Moisture Sorption

**Figure 7:**
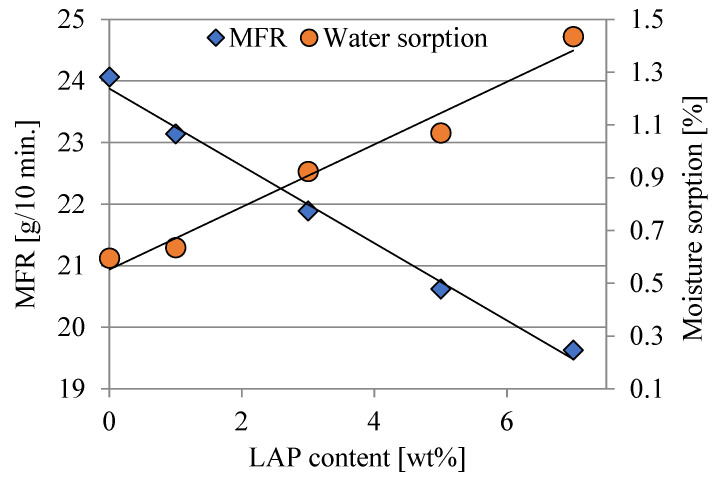
Mass melt flow rate (MFR) and moisture sorption of samples studied.

Due to the application of a type of PBS with a relatively high MFR value, suitable for injection molding, as well as LAP characterized by a high moisture sorption, changes in the MFR and the moisture sorption of individual composites were determined. Both measured parameters change almost rectilinearly with the increase in LAP content (Figure 7). The MFR values decrease by a maximum of ca. 18%; however, the decrease in melt flow from ca. 24.0 to ca. 19.5 g/10 min should not have a significant impact on the processing of this type of composites. This process, in turn, will be significantly influenced by the presence of moisture. The data obtained shows that moisture sorption increases by a maximum of 140%. This is mainly due to the extremely easy sorption of moisture by LAP [52,53]. Due to this phenomenon, the composites produced should be dried before further processing to prevent possible product defects or hydrolysis and degradation of the material during processing.

## 4. Conclusions

The developed PBS/LAP composites are characterized by a good filler distribution in the polymer matrix, and acceptable adhesion at the polymer-filler interface. It was also found that micrometric-sized, practically unchanged LAP particles were present in the matrix, which means that this filler was not fragmented during the extrusion. The filler used only causes some thermal properties of PBS to change, mainly the crystallization rate and the form of the crystallites created. LAP significantly decreases the impact strength and elongation at break of PBS and increases its elastic modulus. However, the strength of the developed composites changes less with an increase in the LAP content, i.e., it slightly decreases during tensile tests or slightly increases during bending tests. The filler used also lowers the melt flow rate of PBS and significantly increases moisture sorption. Changes in the properties of the PBS presented are mainly due to the structure of the LAP applied, which, on the one hand, was not intercalated and, on the other hand, was a modifier with micrometer-sized particles that did not change their structure in the processing stage compared to the original LAP. The developed composites can be widely used, especially in the production of new and fully biodegradable packaging, including flexible and rigid food packaging. Their susceptibility to biodegradation can be controlled to some extent by using different amounts of LAP, which easily absorbs moisture and thus can accelerate the initiation of this process.

## Figures and Tables

**Table 1:** Summary of DSC parameters (T_cc_—cold crystallization temperature, ΔH_cc_—cold crystallization enthalpy, T_m_—melting temperature, ΔH_m_—melting enthalpy, T_c_—crystallization temperature, ΔH_c_—crystallization enthalpy).

Sample	T_cc_ [°C]	ΔH_cc_ [J/g]	T_m_ [°C]		ΔH_m_ * [J/g]	T_c_ [°C]	ΔH_c_ [J/g]
			Peak 1	Peak 2			
PBS	87.80	71.27	104.28	114.86	71.26	-	-
PBS/LAP 99/1	86.59	69.54	104.73	115.01	69.21	-	-
PBS/LAP 97/3	83.47	66.31	102.40	114.22	66.21	105.94	0.26
PBS/LAP 95/5	81.20	58.29	96.79	114.31	59.27	104.83	0.95
PBS/LAP 93/7	80.44	63.86	95.91	114.24	64.84	104.28	1.30

* ΔH_m_ = ΔH_m, peak 1_ + ΔH_m, peak 2._

**Table 2:** The extrusion process parameters for PBS and its composites with LAP.

Sample	M [Nm]	P [kW]	T_D_ [°C]
PBS	11–12	0.35–0.36	169–170
PBS/LAP 99/1	11–12	0.36–0.37	169–170
PBS/LAP 97/3	12–13	0.37–0.38	169–170
PBS/LAP 95/5	12–13	0.37–0.39	170–171
PBS/LAP 93/7	13–14	0.38–0.40	170–171

## Data Availability

The original contributions presented in this study are included in this article, further inquiries can be directed to the corresponding author.
